# High Cytoplasmic Expression of Krüppel-like Factor 4 Is an Independent Prognostic Factor of Better Survival in Hepatocellular Carcinoma

**DOI:** 10.3390/ijms15069894

**Published:** 2014-06-03

**Authors:** Hui-Ting Hsu, Pei-Ru Wu, Chih-Jung Chen, Li-Sung Hsu, Chung-Min Yeh, Ming-Tai Hsing, Yi-Shan Chiang, Ming-Tsung Lai, Kun-Tu Yeh

**Affiliations:** 1 Department of Pathology, Changhua Christian Hospital, Changhua 500, Taiwan; E-Mails: javawomanfanny@gmail.com (H.-T.H.); peiruwu5@gmail.com (P.-R.W.); cjchen1016@gmail.com (C.-J.C.); 28935@cch.org.tw (C.-M.Y.); 2 Institute of Medicine, Chung Shan Medical University, Taichuang 402, Taiwan; 3 School of Medicine, Chung Shan Medical University, Taichuang 402, Taiwan; 4 Department of Medical Technology, Jen-Teh Junior College of Medicine, Nursing and Management, Miaoli 356, Taiwan; 5 Institute of Biochemistry and Biotechnology, Chung Shan Medical University, Taichung 402, Taiwan; E-Mail: lsh316@csmu.edu.tw; 6 Clinical Laboratory, Chung Shan Medical University Hospital, Taichung 402, Taiwan; 7 Department of Neurosurgery, Changhua Christian Hospital, Changhua 500, Taiwan; E-Mail: javafatmark@gmail.com; 8 Biobank, Changhua Christian Hospital, Changhua 500, Taiwan; E-Mail: 146688@cch.org.tw; 9 Department of Pathology, Chung Shan Medical University, Taichung 500, Taiwan; E-Mail: mtlai@csmu.edu.tw; 10 Department of Pathology, Chung Shan Medical University Hospital, Taichung 500, Taiwan

**Keywords:** KLF4, Ki-67, hepatocellular carcinoma, survival, tissue microarray, immunohistochemical study

## Abstract

Hepatocellular carcinoma (HCC) is the second leading cause of cancer-related mortality in the world. Hepatocarcinogenesis is complex, with an extraordinary molecular heterogeneity. Krüppel-like factor 4 (KLF4) plays an important role in cell proliferation and differentiation, and it can function as a tumor suppressor or an oncoprotein, depending on tissue type. The role of KLF4 in HCC remains controversial. The aim of this study was to explore the clinical significance of KLF4 expression in HCC. The study included 205 patients with surgical resection. We performed immunostaining for KLF4 and Ki-67 to investigate the correlations of the clinicopathological parameters of HCC and to examine the proliferative index. KLF4 staining was observed in the cytoplasm of non-tumorous hepatocytes and tumor cells. We subdivided the immunohistological staining results for KLF4 into low expression (Staining 0 and 1+) and high expression (Staining 2+ and 3+) subgroups. The expression of KLF4 was significantly correlated with tumor differentiation (*p* = 0.001). The Ki-67 proliferative index was significantly lower in well-differentiated HCCs (0.781% ± 1.02% *vs.* 2.16% ± 3.14%, *p* = 0.012), but not significantly different between low-KLF4 expression and high-KLF4 expression (1.87% ± 2.93% *vs.* 2.51% ± 3.28%, *p* = 0.32). Kaplan–Meier analysis showed that a high expression of KLF4 was significantly correlated with a longer disease-specific survival (*p* = 0.019). Univariate and multivariate analyses showed that high KLF4 expression was an independent predictor of a better disease-specific survival (*p =* 0.017; hazard ratio = 0.398; 95% confidence interval: 0.19–0.85). High cytoplasmic expression of KLF4 was associated with better disease-specific survival and was an independently favorable prognostic factor in hepatocellular carcinoma. These promising results suggest that KLF4 may play an anti-oncogenic role in hepatocarcinogenesis.

## 1. Introduction

Hepatocellular carcinoma (HCC), the most common primary liver cancer, is the fifth most common cancer in men and the seventh in women. HCC is also the second leading cause of cancer-related mortality in the world [1]. The prevalence of HCC is highest in sub-Saharan Africa and Eastern Asia, with incidence rates of more than 20 per 100,000 individuals. Various risk factors have been linked to HCC, including chronic hepatitis B virus (HBV) or hepatitis C virus (HCV) infections, aflatoxin, chronic alcohol abuse and nonalcoholic fatty liver disease (NAFLD) [1,2]. HCC is known to possess an extraordinary genetic complexity and molecular heterogeneity. Hepatocarcinogenesis is a multistep process and is strongly associated with an increase in allelic losses and the accumulation of chromosomal, genetic and epigenetic alterations [3]. Several oncogenes, tumor suppressor genes and signaling pathways are deregulated in HCC development [3,4,5]. However, the most critical pathways and gene involvement in HCC pathogenesis remain incompletely understood.

Krüppel-like factors (KLFs) are characterized by a highly-conserved carboxyl-terminal DNA-binding domain containing three C2H2 zinc fingers, which are similar to the Krüppel protein found in *Drosophila* [6,7]. KLFs bind to specific DNA sequences, including CACCC-boxes and GC-boxes, and regulate cellular proliferation, differentiation, growth, development, apoptosis and responses to external stress [7]. KLFs function as transcriptional activators or repressors and play important roles in normal physiology, pathophysiology and carcinogenesis [6,7].

The KLF family consists of at least 16 different members, and KLF4 is predominantly expressed in terminally-differentiated epithelial cells of the gastrointestinal tract, skin, vascular endothelial cells and thymus [6,7]. KLF4, formerly known as gut-enriched KLF or epithelial zinc finger, can inhibit cell cycle progression by activating cell cycle checkpoints and promoting cellular differentiation [7]. The role of KLF4 has been extensively examined in several types of cancer and has been found to function as a tumor suppressor or an oncoprotein in a tissue type-dependent manner [7]. Decreased or absent KLF4 expression is present in the cancerous part of colorectal carcinoma [7,8], cervical squamous cell carcinoma (SCC) [9], epithelial ovarian cancer [10], pancreatic ductal carcinoma [11], nasopharyngeal carcinoma (NPC) [12], primary lung carcinoma [13,14], bladder cancer [15], gastric cancer [7,16], esophageal SCC [7] and various types of renal cell carcinoma (RCC) [17]. In contrast, activated or upregulated KLF4 expression is present in primary ductal carcinoma of the breast [6], head and neck SCC [6] and skin SCC [18]. However, there are conflicting reports regarding KLF4 expression in tumor cells and its association with overall survival in HCC [19,20]. In this study, we used the immunohistochemical study of tissue microarray to evaluate the expression of KLF4 and the clinical-pathological relationships of HCC patients in Taiwan.

## 2. Results and Discussion

### 2.1. Patient Characteristics

The study group included 121 males and 84 females, ranging in age from 29 to 87 years, with a mean age of 62.2 years and a median age of 65 years. Of these patients, 118 (57.6%) had hepatitis B infection and 79 (38.5%) had hepatitis C infection, while 16 (7.8%) had concurrent hepatitis B and hepatitis C infection. Cirrhosis was clinically diagnosed in 89 (43.4%) patients. Moderately-differentiated (G2) tumors were the most common and were present in 115 patients (56.1%), followed by poorly-differentiated (33.7%) and then well-differentiated (10.2%) tumors. The cohort included 102 patients (49.8%) in Stage I, 57 patients (27.8%) in Stage II, 34 patients (16.6%) in Stage III and 12 patients in Stage IV (5.9%). Tumors recurred in 114 patients (59.1%) during the follow-up period.

### 2.2. KLF4 Expression Is Associated with Tumor Differentiation in Hepatocellular Carcinoma

**Figure 1:**
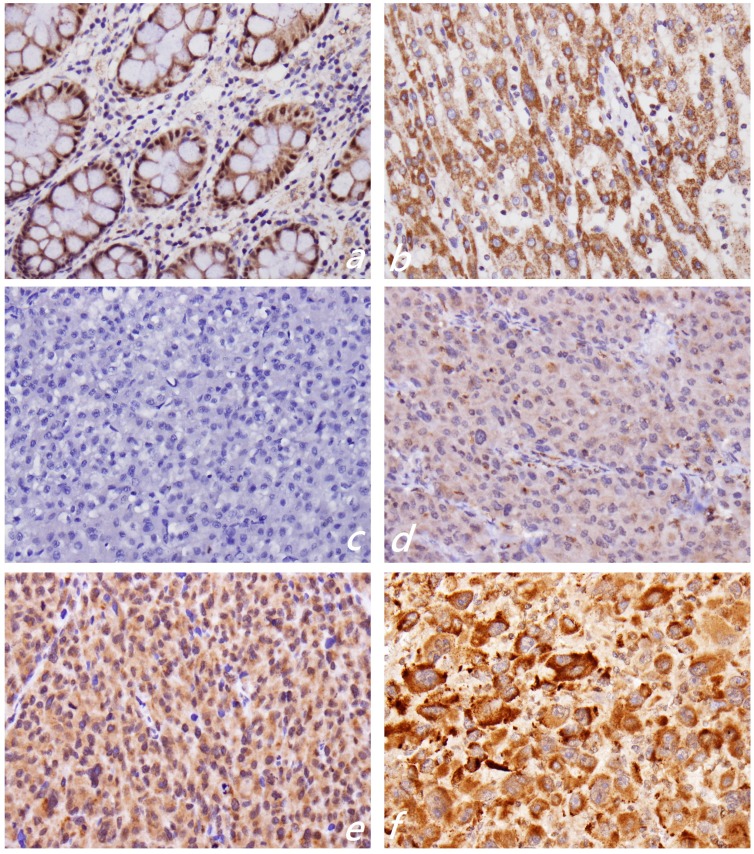
Immunohistochemical staining for the expression of KLF4. Normal colonic mucosa shows nuclear staining (**a**); while non-tumorous hepatocytes show strong cytoplasmic staining (**b**). Based on the relative staining intensity of KLF4 in the cytoplasm, KLF4 staining in hepatocellular carcinoma is classified as 0 (**c**), 1+ (**d**), 2+ (**e**) and 3+ (**f**). We further divide the staining results into low KLF4 (0 and 1+) and high KLF4 (2+ and 3+) subgroups. Original magnification: 400×.

Immunohistochemistry revealed strong KLF4 expression in the cytoplasm of non-tumor or normal hepatocytes (Figure 1b). The staining intensity of KLF4 in non-tumor hepatocytes was used as an internal positive control and provided a scoring baseline for KLF4 staining. The KLF4 was stained in the cytoplasm of the tumor cells. We used the relative staining intensity of KLF4 in the cytoplasm to subdivide the KLF4 immunostaining results into low KLF4 (KLF4 Staining 0 and 1+) (Figure 1c,d) and high KLF4 (KLF4 Staining 2+ and 3+) subgroups (Figure 1e,f). The results revealed 160 patients (78.0%) with low KLF4 expression and 45 patients (22.0%) with high KLF4 expression. We then investigated the associations between cytoplasmic KLF4 expression and the clinicopathological features of HCC. As shown in Table 1, cytoplasmic KLF4 expression was significantly correlated with better tumor differentiation (*p* = 0.001) and a favorable five-year and long-term disease-specific survival rate. No difference was noted in cytoplasmic KLF4 expression when stratified by gender (*p* = 0.411), age (*p* = 0.056), T classification (*p* = 0.520), tumor stage (*p* = 0.366), tumor recurrence (*p* = 0.301) or tumor metastasis (*p* = 0.174).

One known function of KLF4 is that it serves as a transcription factor within the nucleus and induces transformation [21]. A regulated subcellular localization is commonly observed in many transcription factors implicated in neoplasia. For KLF4, a putative SH3 domain binding site near the NH_2_ terminus, is thought to mediate this interaction [21]. Liu *et al.* found that platelet-derived growth factor-BB (PDFG-BB) could induce the translocation of KLF4 to the cytoplasm in vascular smooth muscle cells by the CRM1-mediated nuclear export pathway and that it promoted the interaction of KLF4 with actin in the cytoplasm [22]. In addition, PDGF-BB promoted phosphorylation and SUMOylation of KLF4 to regulate cytoskeletal organization. Cytoplasmic KLF4 expression has been observed in some neoplasias, including colorectal cancer [8], NPC [12], gastric adenocarcinoma [16], and prostate cancer [23]. Decreased cytoplasmic KLF4 expression is significantly correlated with the degree of differentiation in colorectal cancer [8] and significantly associated with shorter overall survival of NPC [12]. In line with previous findings, our results suggested that suppressed KLF4 expression may play an unfavorable role in HCC progression and may serve as a marker for worse differentiation and poor prognosis.

**Table 1:** Patient characteristics and the status of cytoplasmic KLF4 expression by immunohistochemistry.

Variables	Total, *n* (%)	KLF4, *n* (%)		*p*-Value
		KLF4 (−)	KLF4 (+)	
Total number	205 (100)	160 (78.0)	45 (22.0)	
Age				
<62	84 (41.0)	60 (37.5)	24 (53.3)	0.056
≥62	121 (59.0)	100 (62.5)	21 (46.7)	
Gender				
Male	151 (73.7)	120 (75.0)	31 (68.9)	0.411
Female	54 (26.3)	40 (25.0)	14 (31.1)	
Differentiation				
G1	21 (10.2)	11 (6.9)	10 (22.2)	0.001 *
G2	115 (56.1)	99 (61.9)	16 (35.6)	
G3	69 (33.7)	50 (31.3)	19 (42.2)	
T classification				
T1	103 (50.2)	77 (48.1)	26 (57.8)	0.520
T2	59 (28.8)	48 (30.0)	11 (24.4)	
T3–T4	43 (21.0)	35 (21.9)	8 (17.8)	
Stage				
I	102 (49.8)	76 (47.5)	26 (57.8)	0.366
II	57 (27.8)	48 (30.0)	9 (20.0)	
III−IV	46 (22.4)	36 (22.5)	10 (22.2)	
Recurrence				
No	82 (40.0)	61 (38.1)	21 (46.7)	0.301
Yes	123 (60.0)	99 (61.9)	24 (53.3)	
Distant metastasis				
No	198 (96.6)	156 (97.5)	42 (93.3)	0.174
Yes	7 (3.4)	4 (2.5)	3 (6.7)	
Survival				
≤5 years	126 (69.6)	94 (74.6)	32 (25.4)	0.028 *
>5 years	55 (30.4)	49 (89.1)	6 (10.9)	
Disease-specific survival				
Alive	101 (59.4)	74 (73.3)	27 (26.7)	0.017 *
Death	69 (40.6)	61 (88.4)	8 (11.6)	

KLF4 (−): low-KLF4 expression; KLF4 (+): high-KLF4 expression; The *p*-value was measured by the Fisher Exact Test. * *p* < 0.05.

### 2.3. Ki-67 Proliferative Index

High expression of the Ki-67 proliferative index is associated with differentiation grade [24], early disease recurrence and poor prognosis [25]. We used the free web application, ImmunoRatio [26], to assess the Ki-67 proliferative index in the tissue microarray of HCCs. Well-differentiated HCCs had a mean Ki-67 proliferative index of 0.781% ± 1.02%, while this value was 2.16% ± 3.14% in moderately to poorly differentiated HCCs (*p* = 0.012). The Ki-67 proliferative index was significantly correlated with differentiation grade. The mean Ki-67 proliferative index was 1.87% ± 2.93% for low-KLF4 expression and 2.51% ± 3.28% for high-KLF4 expression, but this difference was not statistically significant (*p* = 0.32). These discrepant findings may have resulted from heterogeneous expression of Ki-67 staining and limited representative tissue in the tissue microarray. Further whole-mount tissue sections may be needed to evaluate the association between KLF4 expression and the Ki-67 proliferative index.

### 2.4. Survival Analysis

**Figure 2:**
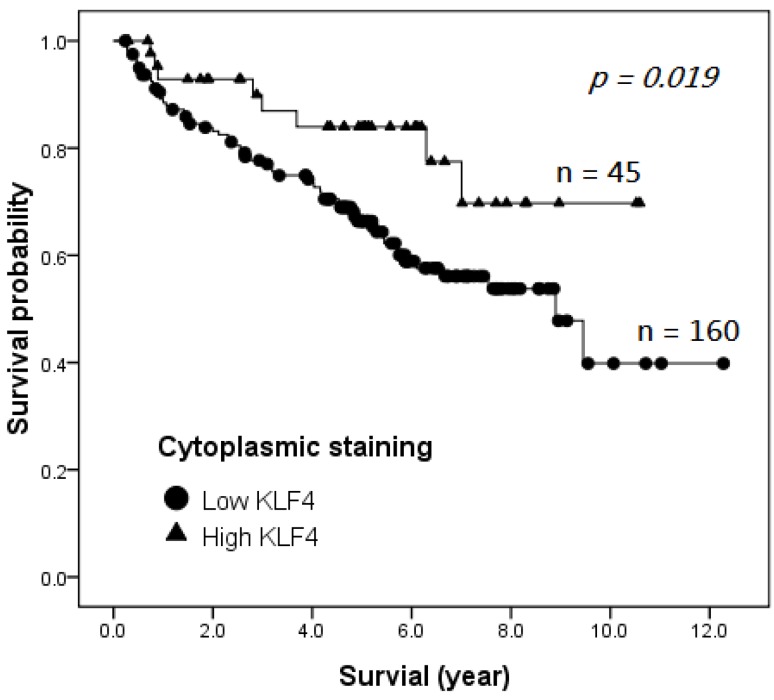
Kaplan–Meier survival curves of disease-specific survival stratified for KLF4 cytoplasmic expression.

Clinicopathologic and outcome information was available for all 205 HCC patients, and 170 of them died of hepatocellular carcinoma during the follow-up period. The patients were followed-up until December 2012, so the follow-up period ranged from 0.2 to 12.3 years, with a mean of 4.6 years. The Kaplan–Meier survival curves of the patients with HCC showed that the patients with high cytoplasmic KLF4 expression had a significantly better disease-specific survival when compared with patients with low cytoplasmic KLF4 expression, as defined by the log-rank test (*p* = 0.019, Figure 2). Univariate analysis indicated that high KL4 expression (*p* = 0.036), tumor differentiation (*p* < 0.001), T classification (*p* < 0.001), recurrence (*p* < 0.001) and distant metastasis (*p* < 0.001) affected the disease-specific survival (Table 2). Multivariate analysis showed that high KLF4 expression remained an independent predictor of a better disease-specific survival (Table 2; *p* = 0.017; hazard ratio = 0.398; 95% confidence interval: 0.19–0.85).

**Table 2:** Univariate and multivariate analysis of the disease-specific survival rate in hepatocellular carcinoma.

Variable	Univariate			Multivariate		
	Hazard Ratio	95% CI	*p*	Hazard Ratio	95% CI	*p*
KLF4 expression						
low	1.0	0.22–0.95	0.036 *	1.0	0.19–0.85	0.017 *
high	0.455			0.398		
Differentiation grade						
G1–G2	1.0	1.30–2.09	0.000 *	1.0	1.32–2.17	0.000 *
G3	1.649			1.686		
T classification						
T1–T2	1.0	1.48–2.43	0.000 *	1.0	1.49–2.50	0.000 *
T3–T4	1.897			1.928		
Distant metastasis						
no	1.0	2.89–15.99	0.000 *	1.0	1.56–9.60	0.003 *
yes	6.793			3.871		
Recurrence						
no	1	1.72–5.97	0.000 *	1	1.35–4.84	0.004 *
yes	3.20			2.556		

* *p* < 0.05.

We used the immunohistochemical technique of tissue microarray in this study to examine the KLF4 distribution in HCC tissue and the clinical-pathological relationships. Cytoplasmic KLF4 expression was lower in HCCs than in non-tumorous hepatocytes. High KLF4 expression was noted in 45 patients (22%). Cytoplasmic KLF4 expression was significantly correlated with tumor differentiation grade (*p* = 0.001). Cellular differentiation is known to be a significant prognostic predictor of survival and recurrence for HCC after various treatments [27,28]. We found that HCC exhibiting high cytoplasmic KLF4 expression tended to have a better differentiation grade and a better five-year, as well as long-term disease-specific survival. Although not statistically significant, a trend was observed for a correlation between low KLF4 expression and higher T classification, advanced stage, tumor recurrence and distant metastasis. Kaplan–Meier analysis showed that high KLF4 expression was significantly correlated with a longer disease-specific survival (*p* = 0.019). Univariate and multivariate Cox regression analyses demonstrated that KLF4 expression, T classification, differentiation grade, distant metastasis and recurrence were independently associated with disease-specific mortality. Consequently, high cytoplasmic KLF4 expression can be considered as an independent predictor of a longer disease-specific survival in HCC patients.

Krüppel-like family proteins play important roles in cellular proliferation, differentiation, growth, development, apoptosis and responses to external stress [7]. KLF4 can act as a tumor suppressor or as an oncoprotein depending on the cellular context [7]. As a tumor suppressor, KLF4 expression is lost or decreased in colorectal carcinoma [7], cervical SCC [9], epithelial ovarian cancer [10], pancreatic ductal carcinoma [11], NPC [12], primary lung carcinoma [7,13], bladder cancer [7,15], gastric cancer [7,16], esophageal SCC [7] and various types of RCC [17]. In colorectal cancer, KLF4 protein expression is significantly correlated with the degree of differentiation and the diagnostic stage [8]. Overexpression of KLF4 has no effect on the proliferation of ovarian cancer cells, but reduced KLF4 expression in ovarian cancers may lead to a reduction in the Bcl-2/Bax ratio [10]. Overexpression of KLF4 in a pancreatic ductal adenocarcinoma cell line induces significant suppression of proliferation by upregulation of p21 and downregulation of cyclin D1 [11]. In NPC, low levels of cytoplasmic KLF4 protein are inversely correlated with the status of N classification and clinical stage [12]. In non-small cell lung cancer, KLF4 expression is significantly inversely correlated with lymph node metastasis and clinical stage [14]. Decreased expression of KLF4 may be associated with advanced tumor stage in gastric cancer [7,16]. Moreover, decreased KLF4 expression is significantly associated with shorter overall survival of NPC, gastric cancer and RCC patients [12,16,17]. The level of KLF4 expression is an independent prognostic indicator of overall survival of NPC and gastric cancer patients [12,16].

On the contrary, levels of KLF4 expression are activated in primary ductal carcinoma of the breast [6,7], in head and neck SCC [6] and in skin SCC [7,18]. Nuclear KLF4 expression in breast cancer cells is a prognostic factor of a shorter overall survival, and it is a marker of an aggressive phenotype in early-stage infiltrating ductal carcinoma [6,7]. Nevertheless, recent studies have revealed discrepant findings in skin SCC and head and neck SCC [29,30,31]. Chen *et al.* [29] showed a uniformly strong nuclear expression in the differentiating and dysplastic squamous epithelium. Their patients with oral SCC and negative nuclear KLF4 expression also had an advanced stage, a high tumor recurrence rate, a poorer three-year survival rate and a 2.5-fold higher risk of death. Tai *et al.* [30] also reported a gradual decline in KLF4 expression between the normal epithelium and most head and neck SCC. However, persistent KLF4 expression in head and neck SCC was associated with a poor prognosis and was an independent predictor of a poorer disease-specific survival [30]. The role played by KLF4 in the carcinogenesis of skin SCC is also complex. Previous studies indicated that increases in KLF4 in the nuclei of basal keratinocytes increase cell turnover and progression and initiate squamous epithelial dysplasia [7,31]. Chen *et al.* [19] reported a significant correlation between the constitutive nuclear KLF4 staining pattern and moderately- and poorly-differentiated SCCs and tumor metastasis. Li *et al.* [31] showed that KLF4 expression, as assayed by immunohistochemistry, was absent in skin SCC when compared with normal tissues. They suggested that KLF4 inhibits cell proliferation, migration and adhesion and that the loss of KLF4 promotes skin tumorigenesis [31].

The role of KLF4 in hepatocarcinogenesis and progression also remains controversial. Li *et al.* [19] demonstrated an intense reduction of KLF4 expression in all human HCC cell lines and human HCC tissues by immunohistochemical study and confirmed these results by real-time polymerase chain reaction (PCR) and western blotting. Furthermore, KLF4 expression was found to be positively correlated with tumor differentiation (*p* < 0.003) and inversely correlated with overall survival rate [19]. That study indicated that KLF4 enhanced VDR expression by binding to the promoter region of VDR and, in turn, synergistically modulating the expression of two cell cycle-regulating proteins, p21 and p27 [19]. However, a more recent study by Yi *et al.* [20] used real-time quantitative PCR analysis to demonstrate that the KLF4 gene expression level was upregulated in HCC tumor tissues as compared with the corresponding adjacent non-tumor liver tissues. They indicated that a high expression of KLF4 was associated with aggressive tumor behavior in terms of vascular invasion and poor tumor differentiation and was independently associated with a poor overall survival and recurrence-free survival [20]. These discrepant results may have arisen due to the different cohorts of samples and techniques used for evaluation.

## 3. Experimental Section

### 3.1. Ethics Statement

The study was approved by the Ethics Committees of Changhua Christian Hospital (Changhua, Taiwan) and adhered to the guidelines approved by the Institutional Review Board (CCH IRB No. 131001). Our institutional Ethics Committees waived the need for consent.

### 3.2. Patients

We enrolled 205 patients who underwent curative operation for hepatocellular carcinoma at the Department of Pathology, Changhua Christian Hospital, Taiwan, from January 1999 to December 2008. Patients who died within 30 days postoperatively or during the postoperative hospital stay were excluded. All of the included patients never received antiviral therapy before or after surgery nor liver transplantation. Tumor clinical staging and histologic differentiation grading were classified according to the criteria of the American Joint Commission on Cancer (AJCC, 7th edition) staging system and the Edmondson and Steiner grading system. G1 tumors were well-differentiated HCCs characterized by mild nuclear atypism, an increased nucleus-to-cytoplasm ratio and a thin trabecular to pseudoglandular arrangement. G2 tumors were moderately-differentiated HCCs with a pseudoglandular or thick trabecular pattern of three or more cell plates. G3 tumors were poorly-differentiated HCCs with solid-to-sheet growth pattern and moderate to marked nuclear pleomorphism, occasionally containing giant or multiple hyperchromatic nuclei. All histopathological and clinical data were obtained, including age, sex, differentiation grade, T classification, stage, recurrence and disease-specific survival.

### 3.3. Tissue Microarray

Representative specimens of hepatocellular carcinoma were selected from hematoxylin- and eosin-stained sections to build a tissue microarray. Tissue cylinders measuring 2 mm in diameter were punched from the marked region of the paraffin block using a homemade semiautomated tissue arrayer. The punched biopsies were obtained from a peripheral or central area with a high number of vital tumor cells and little necrosis. Punches of tumor specimens were arrayed into new paraffin blocks. A 4-µm hematoxylin- and eosin-stained section was reviewed to confirm the presence of morphologically representative lesions of the original cancers.

### 3.4. Immunohistochemistry of KLF4

The immunohistochemical analysis was performed as described. Sections (5-µm) were incubated with anti-KLF4 antibody (1:200 dilution, rabbit polyclonal (sc-20691), Santa Cruz Biotechnology Inc., Heidelberg, Germany) at 4 °C overnight. The Envision™ detection kits (DakoCytomation, K5007, Glostrup, Demark) was used to detect the resulting immune complex, and activity was visualized using aminoethyl carbazole as a substrate. Finally, sections were counterstained with hematoxylin and mounted with Glycergel mounting medium (DakoCytomation). Appropriate positive and negative controls were also included in the same immunohistochemical staining. Two senior pathologists (Hui-Ting Hsu and Pei-Ru Wu) blindly and independently examined each tissue sample and scored according to the intensity of the cytoplasmic staining (no staining: 0; weak staining: 1+; moderate staining: 2+; and strong staining: 3+) and the percentage of immunoreactive cells.

### 3.5. Ki-67 Proliferative Index

The primary antibody was a rabbit monoclonal antihuman Ki-67 antigen (1:200 dilution, SP6, Thermo Scientific, Hertfordshire, UK). The immunohistochemical analysis was performed as described above. Appropriate positive and negative controls were also included in the same immunohistochemical staining. The senior pathologist (Hui-Ting Hsu) took pictures of the region with the greatest density of staining at 200×. We used the free web application, ImmunoRatio (http://153.1.200.58:8080/immunoratio/) [23], to assess the percentage of cells expressing nuclear staining of Ki-67.

### 3.6. Statistical Analysis

Statistical analysis was performed using the Statistical Package for Social Sciences (SPSS) version 17.0 (SPSS, Inc., Chicago, IL, USA). The association between KLF4 the expression and clinicopathologic parameters of HCC was analyzed by the chi-squared or Fisher’s exact test. The two-sample *t*-test was used to compare the Ki-67 proliferative index. For survival analysis, the study end point in our study was disease-specific survival. The Kaplan–Meier estimate was used for survival analysis, and differences between the curves were calculated by the log rank test. The independent prognostic significance, including KLF4 expression, differentiation, T classification, recurrence and distant metastasis, were computed by the multivariate Cox regression model. Differences at *p* < 0.05 were considered to be statistically significant.

## 4. Conclusions

In the current study, high KLF4 expression was noted in 45 patients (22%); this proportion was slightly higher than that reported by Li *et al.* [19], who revealed about 10% of patients with strong KLF4 expression. This may be due to the larger cohort of samples in our study or to different scoring criteria used in the immunohistochemical analysis. In line with the results of Li *et al.*, cytoplasmic KLF4 expression was significantly correlated with better tumor differentiation (*p* = 0.001), favorable five-year disease-specific survival and a longer disease-specific survival (*p* = 0.019). Furthermore, univariate and multivariate Cox regression analyses demonstrated that high cytoplasmic KLF4 expression is an independent predictor of a longer disease-specific survival in HCC patients.

The current study included a large number of HCC patients (205 patients), who were followed-up for a long period (a mean follow-up duration of 4.6 years), and described the immunohistochemical features of cytoplasmic KLF4 expression and its clinical-pathological relationships. One limitation of the study is the lack of further investigation to examine the underlying mechanism driving HCC progression by the loss of KLF4 expression. The study also fails to demonstrate the association of the proliferative index of Ki-67 staining and cytoplasmic KLF4 expression. Additional, well-designed studies are needed in order to elucidate the full function of KLF4 in the tumorigenesis of HCC. In conclusion, the current study indicated that KLF4 potentially serves as a tumor suppressor in HCC development and progression. High KLF4 expression could serve as a favorable prognostic factor of the outcome in patients with HCC.
